# Thermoresponsive bio-affinity interfaces for temperature-modulated selective capture and release of targeted exosomes

**DOI:** 10.1016/j.mtbio.2022.100521

**Published:** 2022-12-11

**Authors:** Kenichi Nagase, Kaichi Yamazaki, Yutaro Maekawa, Hideko Kanazawa

**Affiliations:** Faculty of Pharmacy, Keio University, 1-5-30 Shibakoen, Minato, Tokyo, 105-8512, Japan

**Keywords:** Thermoresponsive polymer, Exosome, Affinity peptide, Polymer brush, Temperature-responsive chromatography

## Abstract

The existing methods for exosome isolation, such as ultracentrifugation, size exclusion, and affinity separation, suffer from some limitations. Herein, we aimed to develop temperature-modulated exosome-capturing materials using thermoresponsive polymers and peptides with affinity for exosomes. Poly(2-hydroxyethyl methacrylate-*co*-propargyl acrylate)-*b*-poly(*N*-isopropylacrylamide) (P(HEMA-*co*-PgA)-*b*-PNIPAAm) was grafted on silica beads *via* a two-step process of activator regenerated by electron transfer atom transfer radical polymerization. Peptides with affinity for exosomes were conjugated to the propargyl group of the bottom P(HEMA-*co*-PgA) segment of the copolymer via a click reaction. The prepared copolymer-grafted beads were characterized by elemental analysis, X-ray photoelectron spectroscopy, scanning electron microscopy, transmission electron microscopy, gel permeation chromatography, and the turbidity of the polymer solution. Results indicated that the copolymer and peptide were successfully modified on the silica beads. Exosomes from SK-BR-3 ​cells, a human breast cancer cell line, were selectively captured on the prepared beads at 37 ​°C, as the upper PNIPAAm segment shrank and the affinity between the peptide and exosome was enhanced. Upon lowering the temperature to 4 ​°C, the captured exosomes were released from the copolymer brush because of the extension of the PNIPAAm segment that reduced the affinity between peptides and exosomes. These findings demonstrated that the prepared copolymer brush-grafted silica beads can capture and release targeted exosomes *via* temperature modulation. Taken together, the developed copolymer brush-grafted silica beads would be useful for the separation of exosomes using simple procedures such as temperature modulation.

## Highlights

• Functional beads were developed using thermoresponsive polymer and affinity peptide.
• Exosome selectively captured on beads through affinity between the peptide and HER2.
• Captured exosome was released from the beads via extension of the polymer.
• Temperature-modulated capture of exosome was achieved via temperature change.

## Graphical abstract

**Figure:**
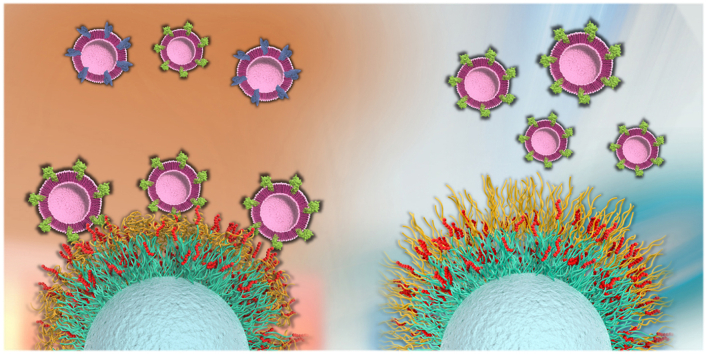

## 1 Introduction

Exosomes are small (40–100 ​nm) vesicles composed of endosomal cell membranes secreted from cells [1]. Recently, exosomes have attracted considerable attention in the biomedical field; since they contain various types of nucleic acids, such as messenger RNA and microRNAs, they have been used as diagnostic markers [2,3]. In addition, exosomes can be used as drug carriers in drug-delivery systems because they have membrane proteins that contribute to specific interactions with cells, thereby facilitating the targeting of specific cells [[4], [5], [6]].

Exosomes need to be purified to be utilized as diagnostic markers and drug carriers. Various methods, including ultracentrifugation, size exclusion, and affinity separation, have been investigated to isolate exosomes and various isolation kits are commercially available (Table S1) [[7], [8], [9], [10], [11], [12], [13], [14], [15], [16], [17], [18], [19]]. However, these separation methods have several limitations. For example, centrifugation and size exclusion methods cannot separate exosomes with membrane- or protein-dependent selectivity, as these methods separate exosomes on the basis of differences in densities and sizes. In addition, exosome separation *via* ultracentrifugation requires large and expensive devices. In contrast, compared to separation methods based on the density and size differences, affinity separation using antibodies exhibits relatively high selectivity. However, for exosome recovery, these methods require the reduction of the affinity between exosomes and antibodies by changing the buffer solution and thus changing properties of exosome membrane proteins. Thus, new exosome purification methods with simple procedures and high selectivity are desired.

The polymer poly(*N*-isopropylacrylamide) (PNIPAAm) shows thermoresponsive hydrophilic and hydrophobic changes, attributed to hydration and dehydration as well as temperature-modulated extension and shrinking [20,21]. PNIPAAm has been successfully applied in drug and gene delivery systems [[22], [23], [24], [25], [26]], biosensors and bioimaging [[27], [28], [29], [30]], nano-actuators [[31], [32], [33], [34], [35]], temperature-responsive chromatography [[36], [37], [38], [39], [40], [41], [42], [43], [44], [45], [46], [47]], cell separation materials [[48], [49], [50], [51], [52], [53], [54], [55], [56]], and cell culture substrates [[57], [58], [59], [60], [61], [62], [63], [64], [65], [66], [67], [68]]. In addition, PNIPAAm has been utilized as a thermoresponsive bioseparation material [[69], [70], [71], [72], [73], [74], [75], [76], [77]]. Bioseparation materials separate proteins, cells, and viral vectors by changing the temperature while maintaining activity, which is attributed to changes in thermoresponsive properties [[69], [70], [71], [72], [73], [74], [75], [76], [77]]. Because these properties of thermoresponsive materials can be exploited to separate exosomes, we focused on PNIPAAm as a new separation tool for exosomes.

In this study, we aimed to develop thermoresponsive exosome-capturing materials using PNIPAAm copolymers and peptides with an affinity for exosomes. Furthermore, we investigated the temperature-modulated exosome capture and release abilities of the prepared copolymer brush-grafted silica beads using exosomes derived from SK-BR-3 ​cells, a human breast cancer cell line.

## 2 Materials and methods

### 2.1 Preparation of thermoresponsive block copolymer brush

**Fig. 1:**
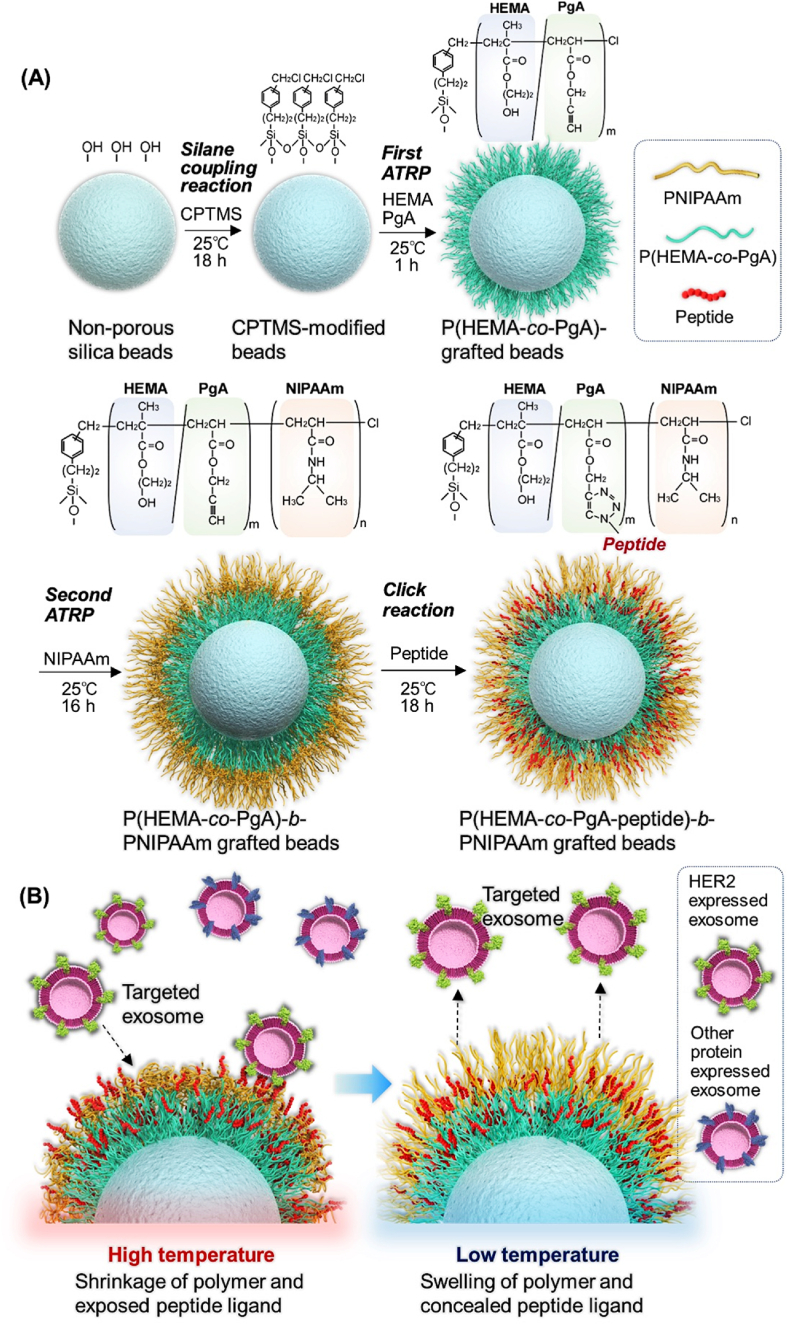
Schematic illustration of (A) preparation of P(HEMA-*co*-PgA)-*b*-PNIPAAm and subsequent modification of affinity peptides with exosomes and (B) temperature-modulated exosome adsorption and desorption.

Thermoresponsive polymer-grafted beads with affinity for exosomes were prepared *via* activators regenerated by electron transfer atom transfer radical polymerization (ARGET ATRP) and subsequent peptide modification was performed *via* a click reaction (Fig. 1A). The materials used for preparation were described in Supplementary Materials. Silica beads were activated with hydrochloric acid at 90 ​°C for 3 ​h. The bead suspension was filtered using a membrane filter; beads were washed with pure water and acetone on a filter membrane, dried at 150 ​°C for 8 ​h, and humidified at 60% relative humidity for 4 ​h ((Chloromethyl)phenylethyl)-trimethoxysilane (CPTMS; 2.7 ​mL, 10.9 ​mmol) was dissolved in 200 ​mL toluene and added to the beads. The silanization reaction was carried out at 25 ​°C for 16 ​h. Thereafter, the bead suspension was filtered through a membrane filter and washed with toluene and acetone. The obtained silica beads were dried at 110 ​°C for 3 ​h.

A poly(2-hydroxyethyl methacrylate-*co*-propargyl acrylate)-*b*-poly(*N*-isopropylacrylamide) (P(HEMA-*co*-PgA)) brush was grafted onto the silica beads *via* the first ATRP of the peptide conjugation segment. HEMA was used as a hydrophilic monomer to suppress the non-specific adsorption of proteins. PgA was used as the site for the click reaction for peptide conjugation because of its propargyl group.

Two compositions of HEMA and PgA (HEMA: PgA ​= ​9:1 or 1:1) were investigated. For preparing HEMA:PgA ​= ​9:1, HEMA (1.17 ​g, 9.00 ​mmol) and PgA (0.11 ​g, 1.00 ​mmol) were added in a mixed solvent of 2-propanol (46 ​mL) and water (4 ​mL). Dissolved oxygen was removed by bubbling with argon gas for 30 ​min. Ascorbic acid (17.6 ​mg, 0.1 ​mmol), Tris[(2-dimethylamino)ethyl]amine (Me_6_TREN; 23.04 ​mg, 0.1 ​mmol), and copper (II) chloride (CuCl_2_) (1.34 ​mg, 0.01 ​mmol) were added to the solution. CPTMS-modified silica beads (3 ​g) and α-chloro-*p*-xylene (1.75 ​μL, 0.0132 ​mmol) were dissolved into the solution under an argon gas atmosphere. ATRP was conducted by continuously shaking the bead suspension at 25 ​°C for 1 ​h. Subsequently, the bead suspension was filtered, and the beads were washed with acetone and dried at 50 ​°C for 3 ​h. The reaction solution was dialyzed, using a membrane filter with a 1 ​kDa molecular weight cut-off, against pure water for 3 days. The purified polymer solution was lyophilized. The obtained P(HEMA-*co*-PgA)-grafted beads were named “H9P1” and “H5P5,” which were synthesized using PgA concentrations of 10 ​mol% and 50 ​mol%, respectively.

The thermoresponsive polymer PNIPAAm was grafted onto the P(HEMA-*co*-PgA) brush *via* a second ATRP. During polymerization, NIPAAm length was modulated by changing the NIPAAm monomer concentration to 500 and 1500 ​mM. For the modification of long PNIPAAm, NIPAAm (8.49 ​g, 75.1 ​mmol) was added to a mixed solvent of 2-propanol (46 ​mL) and water (4 ​mL). Dissolved oxygen was removed by bubbling with argon gas for 20 ​min CuCl_2_ (1.34 ​mg, 0.01 ​mmol), ascorbic acid (17.6 ​mg, 0.1 ​mmol), and Me_6_TREN (23.04 ​mg, 0.1 ​mmol) were added to the solution. P(HEMA-*co*-PgA)-grafted silica beads (1 ​g) and α-chloro-*p*-xylene (1.75 ​μL, 0.0132 ​mmol) were then added to the reaction solution under an argon gas atmosphere. ATRP was performed by continuously shaking the bead suspension at 25 ​°C for 16 ​h. Next, the bead suspension was filtered, and the beads were washed with acetone and dried at 50 ​°C for 3 ​h. The reaction solution was dialyzed as mentioned earlier. The purified polymer solution was lyophilized. The obtained P(HEMA-*co*-PgA)-*b*-PNIPAAm-grafted beads were named as “HxPy-Nz,” where z is the millimolar concentration of NIPAAm in the ATRP.

Peptides with affinity for exosomes were modified to the propargyl group of the copolymer brush via copper-catalyzed azide alkyne cycloaddition (CuAAc), a click reaction [78,79]. Two types of peptides, N_3_-Gly-Gly-Gly-Leu-Thr-Val-Ser-Pro-Trp-Tyr (N_3_-GGGLTVSPWY) and N_3_-Gly-Gly-Gly-Lys-Cys-Cys-Tyr-Ser-Leu (N_3_-GGGKCCYSL), were used as affinity peptides; these peptides are used as peptide imaging agents with HER2 specificity [80] and should, therefore, function as ligands for the capture of HER2-expressing exosomes. Dimethyl sulfoxide (DMSO): H_2_O ​= ​1:1(v/v) was prepared and deoxygenated by bubbling with argon gas. The peptide was dissolved in solvent at a concentration of 2 ​mg/mL and mixed with DMSO:H_2_O ​= ​1:1 (v/v) (2 ​mg/mL, 2 ​mL), Tris[(1-benzyl-1H-1,2,3-triazol-4-yl)methyl]amine DMSO solution (5 ​mM, 2 ​mL), copper (II) sulfate pentahydrate aqueous solution (2.5 ​mM, 2 ​mL), and sodium ascorbate water solution (50 ​mM, 2 ​mL). The prepared P(HEMA-*co*-PgA)-grafted beads (400 ​mg) or P(HEMA-*co*-PgA)-*b*-PNIPAAm-grafted beads (400 ​mg) were then added to the mixture. The click reaction proceeded with the continuous shaking of the bead suspension at 25 ​°C for 18 ​h. The bead suspension was filtered using a membrane filter; beads on the filter were washed with water and acetone and dried *in vacuo* at 25 ​°C for 2 ​h. The peptide-modified beads were named “HxPy-Nz-LTV” and “HxPy-Nz-KCC” and were modified with GGGLTVSPWY and GGGKCCYSL, respectively.

### 2.2 Characterization of copolymer brush-grafted beads with peptides

The polymer-grafted beads were characterized *via* elemental analysis, scanning electron microscopy (SEM), transmission electron microscopy (TEM), X-ray photoelectron spectroscopy (XPS), gel permeation chromatography (GPC), and the turbidity of the polymer solution.

To estimate the amounts of initiator, polymer, and peptide, CHN elemental analysis of the prepared beads was conducted using an elemental analyzer (PE2400-II; PerkinElmer, Waltham, MA, USA). The equations for the estimation are described in the Supplementary Materials.

The surface elemental composition of the P(HEMA-*co*-PgA)-*b*-PNIPAAm brush with affinity peptides was observed using XPS (Quantera SXM, ULVAC-PHI, Kanagawa, Japan). The bead surface was observed using TEM (TecnaiSpirit TEM; Hillsboro, OR, USA), and bead morphology was observed using SEM (TM4000Plus-II; Hitachi High-Tech, Tokyo, Japan).

The molecular weight of the polymer was determined by GPC (HLC-8320GPC EcoSEC; Tosoh, Tokyo, Japan), with two serially connected GPC columns (TSK-GEL α-M; Tosoh) using *N*,*N*-dimethylformamide with 50 ​mM lithium chloride as the mobile phase.

The phase transition behavior of the polymer was observed from the temperature-dependent turbidity change of the polymer solution. The polymer solution (5 ​mg/mL) was prepared using phosphate-buffered saline (PBS). The transmittance of the polymer solution at 600 ​nm was observed with increasing temperature (0.1 ​°C/min).

### 2.3 Temperature-modulated selective capture of exosomes

The temperature-modulated capture of exosomes was performed using the prepared copolymer-grafted beads with affinity peptides. Exosomes were obtained from the culture medium of SK-BR-3 and HeLa cells. SK-BR-3 ​cells or HeLa cells were cultured in McCoy's 5A (Modified) medium containing 10% FBS, 1% NEAA, and 1% PS. Ordinarily, FBS also contains exosomes, leading to the contamination of cell-derived and FBS-derived exosomes. To remove exosomes from FBS, FBS was subjected to ultracentrifugation at 100,000×*g* and 4 ​°C for 18 ​h, and the supernatant was used for the cell culture. We confirmed that the prepared culture medium did not contain exosomes by using dynamic light scattering (DLS; Zetasizer Nano ZS; Malvern Panalytical, Malvern, UK).

Cells were rinsed using the prepared cell culture medium. To produce exosomes, these cells were cultured at 37 ​°C for 72 ​h. Subsequently, 30 ​mL culture medium was collected and centrifuged at 2000×*g* for 30 ​min. The culture supernatant was collected, and half the volume of Total Exosome Isolation kit was added. The medium was then gently stirred and incubated at 4 ​°C for 18 ​h. Next, the suspension was centrifuged at 10,000×*g* and 4 ​°C for 60 ​min. The supernatant was then removed, and 500 ​μL of PBS was added. The suspension was subjected to centrifugation filtration using a 0.2 ​μm-pore diameter filter and the filtrate was used as an exosome suspension. The exosomes obtained in the suspension were confirmed *via* DLS (Zetasizer Nano ZS; Malvern Panalytical, Malvern, UK) to observe particles with 30–100 ​nm diameter. Ordinarily, exosome characterization is performed by confirming various exosome markers such as CD9, CD63, and CD81 [81,82]. In this study, we observed the expression of CD63 and HER2 on exosomes both SK-BR-3 ​cells and HeLa cells *via* western blotting.

For observing temperature-modulated capture of exosomes on the prepared beads, protein concentration of the prepared exosome suspension was measured using the BCA protein assay to determine the exosome amount. Copolymer-grafted beads (10 ​mg or 30 ​mg) were added to a 1.5-mL microtube. A small amount of PBS (0.10 ​mL) was added to condition the beads in PBS, and the beads were incubated at 37 ​°C or 4 ​°C for 10 ​min. After incubation, the exosome suspension was added to the bead suspension to adjust the total protein concentration to 120 ​μg/mL and a volume of 1.0 ​mL. The bead suspension was incubated at 37 ​°C or 4 ​°C for 60 ​min with stirring every 10 ​min for capturing exosome. Next, the bead suspension was centrifuged at 14,000×*g* at 37 ​°C or 4 ​°C for 10 ​min. The supernatant was then collected. RIPA buffer was added to the supernatant, and the solution was sonicated at 4 ​°C for 15 ​min. Protein concentration of the solution was determined using the BCA protein assay. The capture rate of exosomes was calculated as the ratio of the captured exosome amount to the exosome amount added to the beads, which was estimated from the protein concentration. The exosome marker proteins were identified *via* western blotting of the collected supernatant.

For determining temperature-modulated capture and release of exosomes, exosomes were captured on the beads using the same procedure described above at 37 ​°C. Then, the uncaptured exosomes were removed, 1 ​mL PBS warmed at 37 ​°C was added to the beads, and the bead suspension was incubated at 37 ​°C for 10 ​min. The mixture was centrifuged at 14,000×*g* at 37 ​°C for 10 ​min and the supernatant was removed. To collect exosomes from the beads, 1 ​mL PBS cooled at 4 ​°C was added, and the bead suspension was incubated at 4 ​°C for 60 ​min, with gentle stirring every 10 ​min. The incubation time of 60 ​min is sufficient for extension of PNIPAAm segment and releasing exosome. Next, the bead suspension was centrifuged at 14,000×*g* at 4 ​°C for 10 ​min. The supernatant was collected. RIPA buffer was added to the supernatant, and the solution was sonicated at 4 ​°C for 15 ​min. Protein concentration of the solution was determined using the BCA protein assay. The release rate of exosomes was calculated as the ratio of collected exosomes to captured exosomes, which was estimated from the protein concentration. Size distribution of the collected exosomes was determined *via* DLS.

## 3 Results and discussion

### 3.1 Characterization of the copolymer-grafted beads with peptides

CHN elemental analysis was performed to estimate the amount of the modified ATRP initiator, polymer, and peptide on the beads (Table 1). The obtained P(HEMA-*co*-PgA)-grafted beads were named as “H9P1” and “H5P5” and were synthesized using PgA concentrations of 10 ​mol% and 50 ​mol%, respectively. The obtained P(HEMA-*co*-PgA)-*b*-PNIPAAm-grafted beads were named as “HxPy-Nz,” where z is the millimolar concentration of NIPAAm in the ATRP. The peptide-modified beads were named “HxPy-Nz-LTV” and “HxPy-Nz-KCC” and were modified with GGGLTVSPWY and GGGKCCYSL, respectively.

**Table 1:** Characterization of the prepared thermoresponsive block copolymer brush-grafted silica beads.

Samplea)	Elemental composition (%)b)		%C (calcd)c)	Immobilized initiator (μmol/m^2^)d)	Grafted polymer (mg/m^2^)d)	Immobilized peptide (μg/m^2^)d)
	Carbon	Nitrogen				
Unmodified silica beads	0.26 ​± ​0.06	0.18 ​± ​0.02				
CPTMS-modified silica beads	1.42 ​± ​0.03	0.12 ​± ​0.00	36.2	3.61		
H9P1	2.01 ​± ​0.02	0.18 ​± ​0.02	56.2		0.241	
H9P1-N500	2.26 ​± ​0.04	0.23 ​± ​0.02	63.6		0.329	
H9P1-N500-LTV	2.37 ​± ​0.03	0.28 ​± ​0.02	54.7			47.4
H5P5	1.88 ​± ​0.04	0.20 ​± ​0.01	60.0		0.175	
H5P5-N500	2.10 ​± ​0.01	0.25 ​± ​0.04	63.6		0.255	
H5P5-N500-LTV	2.22 ​± ​0.01	0.29 ​± ​0.02	54.7			47.2
H5P5-N1500	2.30 ​± ​0.02	0.28 ​± ​0.02	63.6		0.324	
H5P5-N1500-LTV	2.41 ​± ​0.02	0.32 ​± ​0.00	54.7			47.3
H5P5-LTV	2.04 ​± ​0.06	0.28 ​± ​0.02	54.7			72.0
H5P5-KCC	1.96 ​± ​0.04	0.07 ​± ​0.02	47.0			46.6

a The sample code was determined using the initial feed monomer ratio of 2-hydroxyethyl methacrylate and propargyl acrylate and the molar concentration of *N*-isopropylacrylamide in atom transfer radical polymerization.

b Determined via elemental analysis. Data are mean values with standard deviation (n = 3).

c %C(calcd) is the theoretical carbon percentage of the modified CPTMS or polymer.

d Calculated from the carbon composition.

The carbon percentage of the measured value through elemental analysis exhibited a greater difference compared to the theoretical value, because the weight of the modified polymer on silica beads is lower than that of the silica beads.

Carbon on the ATRP initiator, CPTMS, modified silica beads exhibited a higher concentration of unmodified silica beads, which indicated that the silanization reaction successfully modified the CPTMS on the silica beads. The carbon composition of the CPTMS-modified beads was slightly lower (1.42%) than that of the CPTMS-modified beads (approximately 4%) reported in a previous study [83]. This is because the silica beads in the present study had a non-porous structure and a relatively small surface area. In contrast, the silica beads in the previous study had a porous structure and a large surface area. Thus, the CPTMS-modified silica beads showed a low carbon composition. The amount of CPTMS on the silica beads calculated from the carbon composition and surface area of the silica beads was 3.61 ​μmol/m^2^, which was similar to the value reported in a previous study (approximately 4 ​μmol/m^2^) [83]. These results indicated that CPTMS was modified on the silica beads *via* a silanization reaction with high density, similar to that in a previous report [83]. The P(HEMA-*co*-PgA)-grafted silica beads, H9P1, and H5P5 showed a higher carbon composition than the CPTMS-modified silica beads, indicating that P(HEMA-*co*-PgA) was grafted onto the silica beads *via* the first ATRP. Higher carbon composition was observed on the P(HEMA-*co*-PgA)-*b*-PNIPAAm-grafted silica beads compared to the P(HEMA-*co*-PgA)-grafted silica beads. This result indicated that the PNIPAAm segment was successfully grafted on the P(HEMA-*co*-PgA)-grafted silica beads via the second ATRP. H5P5-N1500 exhibited a higher carbon composition than H5P5-N500, indicating that a larger amount of polymer was grafted on H5P5-N1500 than on H5P5-N500. This is because the long PNIPAAm segment was grafted on H5P5-N1500 compared to H5P5-N500 owing to the high monomer concentration in the ATRP of NIPAAm. A previous report indicated that longer PNIPAAm molecules are grafted on the substrate with an increasing NIPAAm concentration in ATRP [84]. Thus, in the present study, long PNIPAAm was grafted on H5P5-N1500 compared with that on H5P5-N500, leading to a larger amount of copolymer on H5P5-N1500. The peptide-modified beads (e.g., H5P5-N1500-LTV) showed a higher carbon concentration than the beads before peptide modification (e.g., H5P5-N1500). This result indicated that the peptide was successfully modified on the copolymer on the silica beads via the click reaction.

**Fig. 2:**
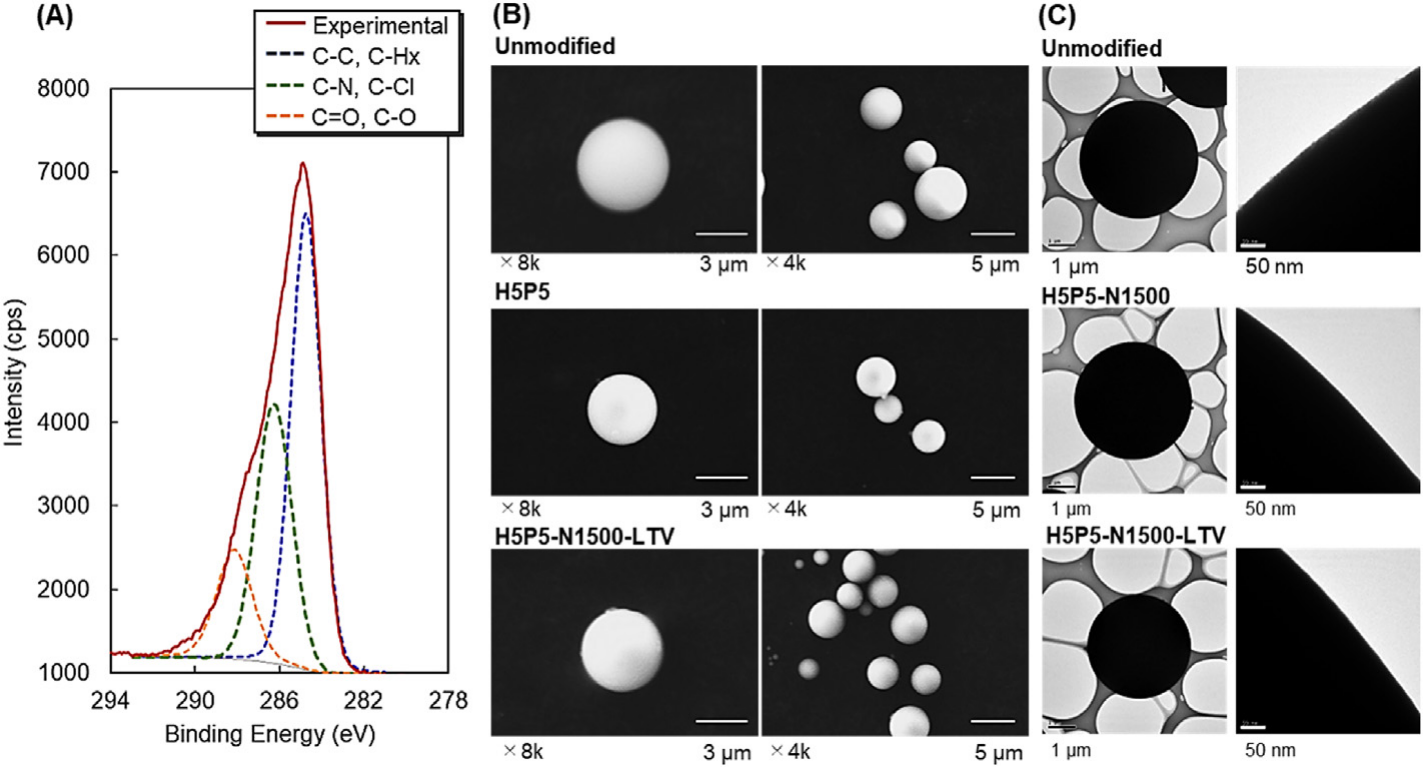
Characterization of the prepared beads. (A) X-ray photoelectron spectroscopic spectra of C1s peak deconvolution of P(HEMA-*co*-PgA)-*b*-PNIPAAm with GGGLTVSPWY (H5P5-N1500-LTV)-modified silica beads, at take-off angle of 90°, (B) Scanning electron microscopy images of the prepared beads and (C) and transmission electron microscopy images of the prepared beads.

The surface elemental compositions of the copolymer and peptide-modified silica beads were determined using XPS (Fig. 2A, Fig. S1, and Table 2). High peak intensity of the C1s peak and carbon composition, i.e., 66.6%, were observed (Fig. S1, Table 2). These results indicated that the silica bead surface was covered by P(HEMA-*co*-PgA)-*b*-PNIPAAm. C1s peak deconvolution of P(HEMA-*co*-PgA)-*b*-PNIPAAm with GGGLTVSPWY-modified silica beads (H5P5-N1500-LTV) was performed to investigate the composition of the carbon bond types (Fig. 2A; each deconvoluted bond is expressed as a dotted line). Peaks attributed to the carbonyl bond were observed at approximately 288 ​eV because P(HEMA-*co*-PgA)-*b*-PNIPAAm with carbonyl bonds was grafted onto the beads. A small amount of chlorine (0.2%) was observed in P(HEMA-*co*-PgA)-*b*-PNIPAAm with GGGLTVSPWY (Table 2). This is because the terminal chlorine of the copolymer was detected with XPS. A previous report indicated that the polymer-grafted silica beads prepared with ATRP exhibit a small amount of chlorine because the terminal chlorine of the polymer is buried inside the polymer brush and some part of the chlorine is lost during ATRP [85]. In a similar manner, the prepared polymer brush on silica beads exhibited a small amount of chlorine in this study.

**Table 2:** Elemental analysis of P(HEMA-*co*-PgA)-*b*-PNIPAAm with GGGLTVSPWY (H5P5-N500-LTV)-modified silica beads *via* X-ray photoelectron spectroscopy at a take-off angle of 90°.

Code	Atom (%)					N/C ratio
	C	N	O	Si	Cl	
P(HEMA-*co*-PgA)-*b*-PNIPAAm with GGGLTVSPWY (H5P5-N1500-LTV)-modified silica beads	66.6	8.8	19.2	5.2	0.2	0.132
						
NIPAAma)	75.0	12.5	12.5	-	-	0.167
HEMAa)	66.7	-	33.3	-	-	-
PgAa)	75.0	-	25.0	-	-	-
Silica beadsa)	-	-	66.7	33.3	-	-
CPTMSa)	70.6	-	17.6	5.88	5.88	-

a Theoretical atomic composition of each monomer, silica beads, and ATRP initiator.

The silica bead morphology was observed using SEM (Fig. 2B and Fig. S2). The silica beads retained their spherical shape without any deformation or aggregation after each reaction step. These results indicated that a series of reactions, including the silanization reaction for CPTMS modification, the first ATRP for modifying P(HEMA-*co*-PgA), the second ATRP for modifying the PNIPAAm segment, and the click reaction for modifying the peptide, did not deform the silica beads. A previous report indicated that PNIPAAm agglomeration is observed on the surface of silica beads when a large amount of PNIPAAm is grafted on silica beads via ATRP under uncontrollable conditions [86]. In contrast, the prepared silica beads grafted with PNIPAAm copolymer exhibited a smooth surface (Fig. 2B). These results indicate that the first and second ATRPs in this study provided a uniform coating of silica beads with the copolymer.

The surface morphologies of the unmodified silica, P(HEMA-*co*-PgA)-*b*-PNIPAAm-grafted silica beads (H5P5-N1500), and P(HEMA-*co*-PgA)-*b*-PNIPAAm with peptide (H5P5-N1500-LTV) grafted beads were observed via TEM (Fig. 2C and Fig. S3). The unmodified beads, copolymer-grafted beads, and copolymer with peptide modification exhibited a smooth surface (Fig. 2C), attributed to the non-porous structure of silica beads in the study. In a previous study, porous silica beads exhibited a rough surface owing to their porous structure [76]. In contrast, the silica beads used in this study had a non-porous structure. Thus, a smooth surface was observed on unmodified silica beads. After modifying the copolymer and peptide, a smooth surface was maintained (Fig. 2C), probably because the thin copolymer layer with a well-defined polymer brush was grafted through ATRP. A previous report indicated that PNIPAAm copolymer modification through conventional radical polymerization exhibits a rough surface in the TEM image, which is attributed to the uncontrolled polymerization of the PNIPAAm copolymer [87]. In contrast, in this study, the PNIPAAm copolymer was grafted through ATRP, leading to controlled polymerization and modification of thin and smooth polymer layers on silica beads.

The molecular weights and polydispersities of the prepared copolymers were characterized via GPC (Table S2). P(HEMA-*co*-PgA), prepared using a monomer feed composition of HEMA and PgA of 1:1, exhibited larger polydispersity than the P(HEMA-*co*-PgA) prepared using a monomer feed composition of HEMA and PgA of 9:1. These results indicated that the polymerization control of PgA in ATRP was weaker than that of HEMA. The PNIPAAm prepared using a high NIPAAm concentration (1500 ​mM) exhibited a large molecular weight and polydispersity than that prepared using a low NIPAAm concentration (500 ​mM). A previous report indicated that the molecular weight of PNIPAAm increases with increasing NIPAAm concentration because of the increased polymerization rate and increased ratio of monomer to the initiator [84]. In a similar manner, long PNIPAAm was synthesized in ATRP using a relatively high monomer concentration (1500 ​mM).

The phase transition behavior of short-chain (N500) and long-chain (N1500) PNIPAAm in PBS was observed (Fig. S4). The short PNIPAAm exhibited a slightly higher phase transition temperature than the long PNIPAAm. A previous report indicated that the phase transition temperature of PNIPAAm with a hydrophobic end group decreases with increasing molecular weight because the effect of the hydrophobic end group on the phase transition increases at short molecular weights [88,89]. In the present study, α-chloro-*p*-xylene was used as an ATRP initiator. Thus, the hydrophobic end group reduced the phase transition temperature of the short PNIPAAm. N500 and N1500 exhibited phase-transition temperatures between 4 and 37 ​°C. These results indicate that the copolymer phase transition could be modulated by changing the temperature from 37 ​°C to 4 ​°C.

### 3.2 Temperature-modulated capture and release of exosomes

Next, the exosome-capturing ability of the prepared beads was investigated. Exosomes were obtained from the culture media of SK-BR-3 and HeLa cells. The particle diameter distribution of the prepared exosome suspension was observed via DLS (Fig. S5). Both SK-BR-3 and HeLa cell suspensions exhibited a size distribution with a peak size of approximately 60 ​nm. A previous report indicated that exosomes have a size distribution of 30–100 ​nm [90]. Thus, our results indicated that exosomes could be obtained from the culture medium of SK-BR-3 and HeLa cells. HER2 and CD63 expression in exosomes was observed using western blotting (Fig. S6). Exosomes from both SK-BR-3 and HeLa cell culture media exhibited CD63 expression. A previous report indicated that exosomes, as a marker, exhibit CD63 expression [90]. Accordingly, our results confirmed that the obtained suspension contained exosomes. Exosomes from SK-BR-3 ​cells exhibited higher expression of HER2 than HeLa cells because SK-BR-3 ​cells express HER2 to a greater extent than HeLa cells. The results indicated that exosomes with different levels of HER2 expression were obtained from SK-BR-3 and HeLa cell culture media.

**Fig. 3:**
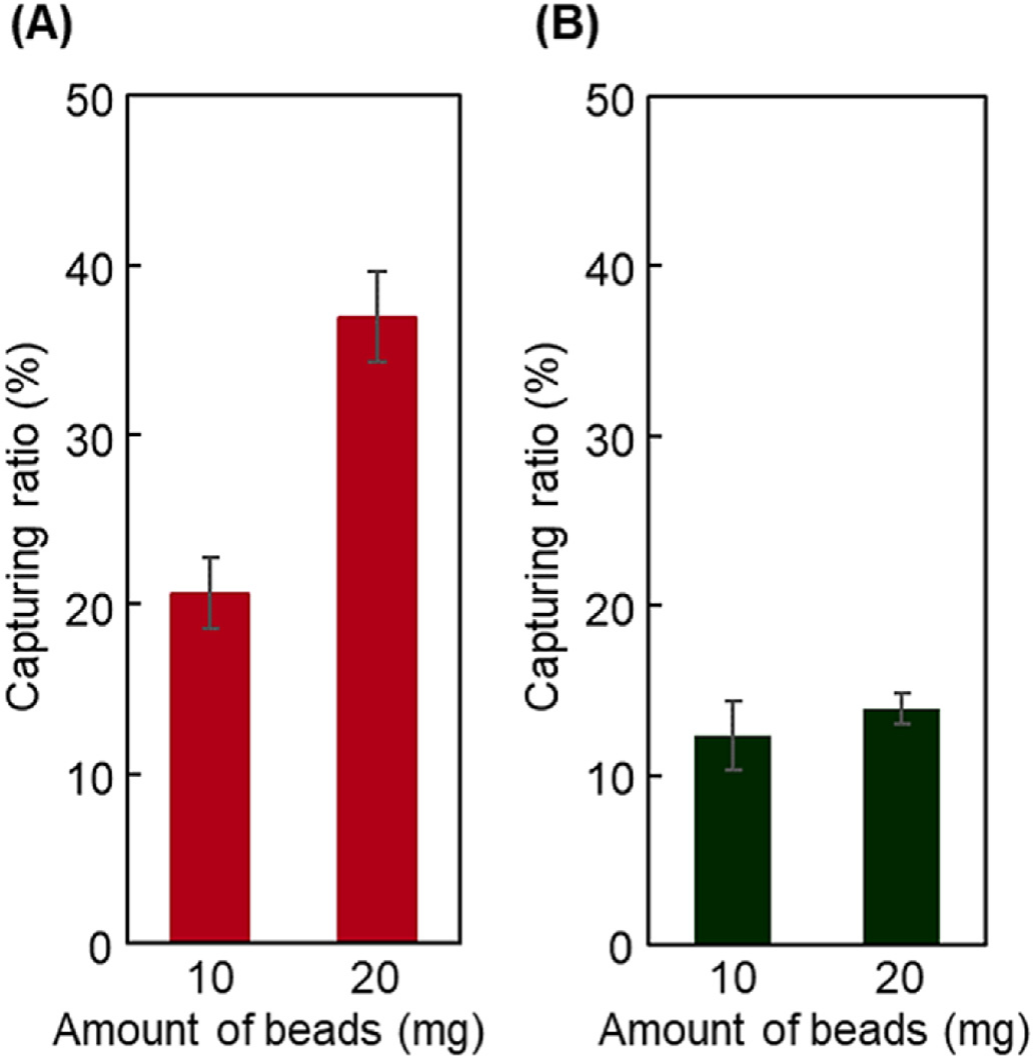
Capturing ratio of exosomes of (A) H5P5-LTV and (B) H5P5-KCC beads. Data are presented as the mean ​± ​standard deviation (n ​= ​3).

To investigate the affinity of peptides for exosomes, two types of peptides, GGGLTVSPWY and GGGKCCYSL, were modified on the P(HEMA-*co*-PgA)-modified beads, and the exosome-capturing ratio was observed using exosomes from SK-BR-3 ​cells (Fig. 3). The GGGLTVSPWY-modified beads H5P5-LTV exhibited a higher capture ratio of exosomes than the GGGKCCYSL-modified beads H5P5-KCC. These results indicated that GGGLTVSPWY was more suitable as a peptide ligand for capturing exosomes than GGGKCCYSL. A previous report suggested that the dissociation constant *K*_d_ of LTVSPWY and HER2 is 4.3 ​nM, whereas that of KCCYSL and HER2 is 295 ​nM [80]. In a similar manner, in the present study, the GGGLTVSPWY-modified beads H5P5-LTV exhibited a stronger affinity for the exosomes derived from SK-BR-3 ​cells than that exhibited by the GGGKCCYSL-modified beads H5P5-KCC. These results indicated that the GGGLTVSPWY-modified beads H5P5-LTV were suitable for capturing HER2-expressing exosomes.

**Fig. 4:**
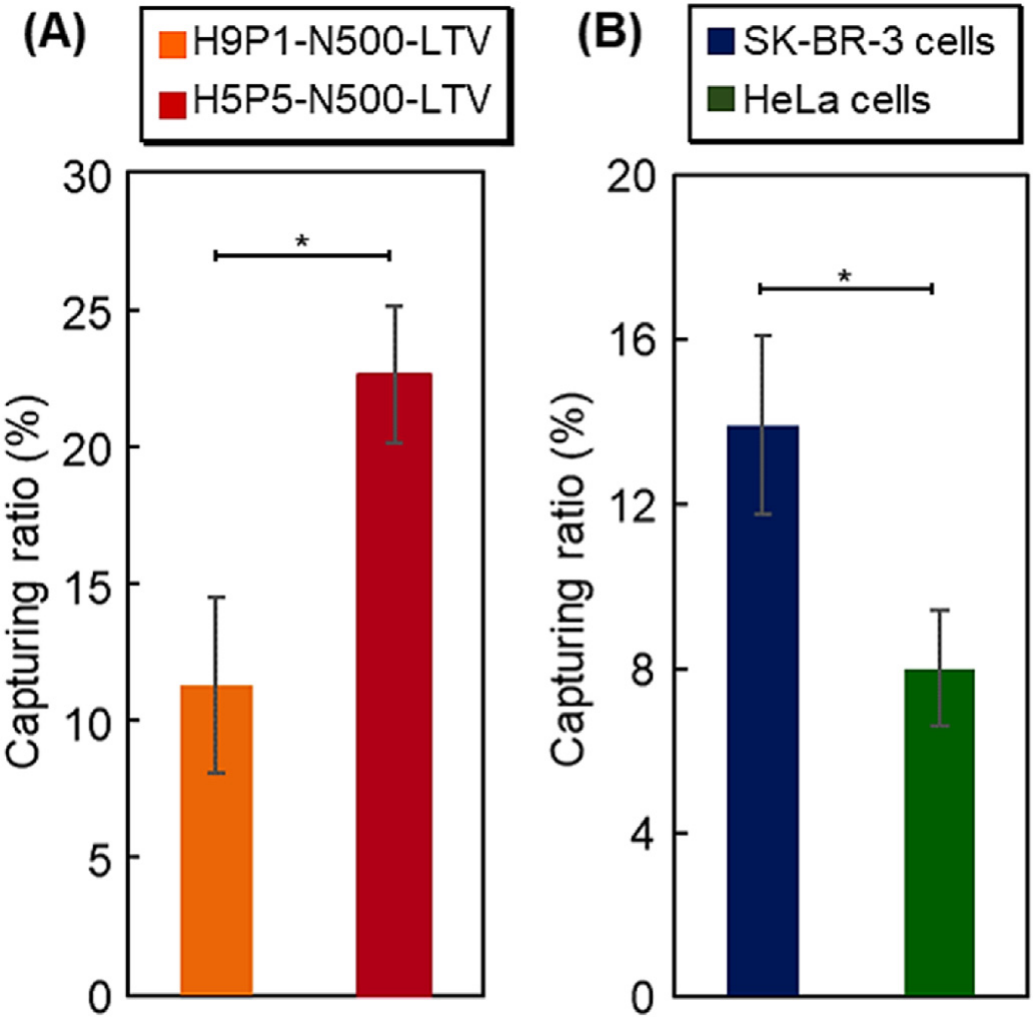
Capturing ratio of exosomes (A) using H9P1-N500-LTV and H5P5-N500-LTV beads and (B) derived from SK-BR-3 and HeLa cells of H5P5-N1500-LTV beads Data are presented as the mean ​± ​standard deviation. (∗*P* ​< ​0.05; n ​= ​3).

To investigate the accurate comonomer composition of P(HEMA-*co*-PgA), the exosome-capturing ratio was observed using two copolymer compositions, H9P1-N500-LTV and H5P5-N500-LTV (Fig. 4A). H5P5-N500-LTV exhibited a higher exosome-capturing ratio than H9P1-N500-LTV. H5P5-N500-LTV has a larger propargyl group than H9P1-N500-LTV, leading to a greater extent of peptide modification on H5P5-N500-LTV. Thus, H5P5-N500-LTV would have a stronger affinity for exosomes than H9P1-N500-LTV, leading to a large capture ratio for exosomes.

To investigate the capture selectivity of the HER2-positive exosomes of the prepared peptide copolymer brush grafted beads, the exosome-capturing ratio of SK-BR-3 and HeLa cells obtained using H5P5-N1500-LTV was observed (Fig. 4B). The SK-BR-3 cell-derived exosomes exhibited a higher capture ratio than that exhibited by exosomes derived from HeLa cells. A previous study indicated that SK-BR-3-derived exosomes express high levels of HER2 [91]. In the present study, western blot analysis revealed that the exosomes derived from SK-BR-3 ​cells exhibited increased expression of HER2 compared to those derived from HeLa cells (Fig. S6). Thus, the prepared H5P5-N1500-LTV captured exosomes derived from SK-BR-3 ​cells to greater extent than those from HeLa cells. These results demonstrate that the prepared H5P5-N1500-LTV could selectively capture exosomes with high HER2 expression, leading to the separation of HER2-overexpressing exosomes from exosomes with low HER2 expression. Furthermore, previous report have indicated that HER2-overexpressing exosomes can be used in breast cancer diagnosis [92]. Thus, the prepared H5P5-N1500-LTV has potential diagnostic applications.

**Fig. 5:**
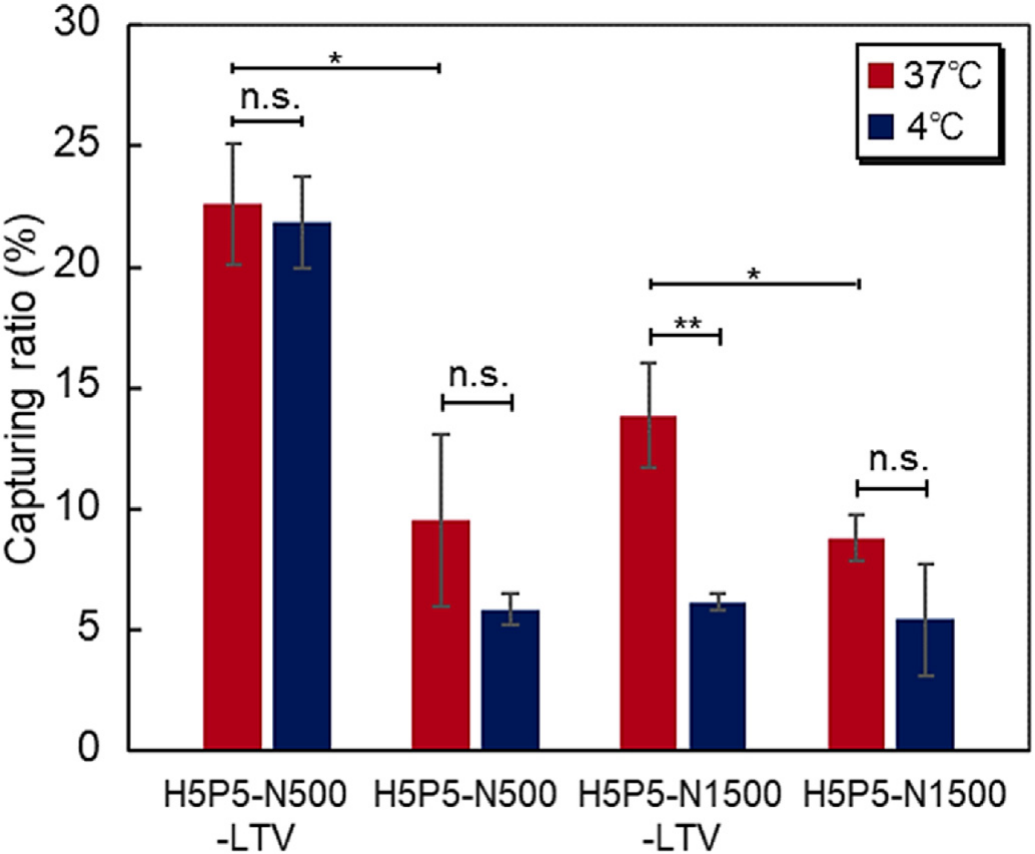
Temperature-dependent capturing ratio of exosomes with different lengths of the PNIPAAm segment and with and without peptide modification. Data are presented as the mean ​± ​standard deviation. (∗*P* ​< ​0.05; ∗∗*P* ​< ​0.01; n. s.: not significant; n ​= ​3).

The effect of PNIPAAm segment length and peptide affinity with exosomes derived from SK-BR-3 ​cells was investigated using two segment lengths of PNIPAAm, with and without peptide modification (Fig. 5). Upon comparing H5P5-N500-LTV and H5P5-N500 at 37 ​°C, H5P5-N500-LTV exhibited a higher exosome-capturing ratio than that exhibited by H5P5-N500. This is because H5P5-N500-LTV has peptides with affinity for exosomes, whereas H5P5-N500 does not. These results also indicated that the PNIPAAm-induced hydrophobicity of H5P-N500 was not effective for capturing exosomes. H5P5-N500-LTV did not exhibit a significant difference in the capture ratios between 37 ​°C and 4 ​°C. This was due to the short PNIPAAm segment of H5P5-N500-LTV. The PNIPAAm segment of H5P5-N500-LTV was relatively short, leading to a small structural change in the PNIPAAm segment owing to temperature-dependent shrinkage and extension. Thus, the affinity between the peptide on the bottom P(HEMA-*co*-PgA) and exosomes was not effectively modulated by a change in temperature. In contrast, H5P5-N1500-LTV exhibited a large difference in the exosome-capturing ratio between 37 ​°C and 4 ​°C. This was attributed to the long PNIPAAm chain of H5P5-N1500-LTV. H5P5-N1500-LTV has a long PNIPAAm segment on the peptide-modified P(HEMA-*co*-PgA). At 37 ​°C, the PNIPAAm segment of the copolymer dehydrated and shrunk, leading to an enhanced affinity between the modified peptide on the bottom copolymer brush and the exosome. In contrast, at 4 ​°C, the PNIPAAm segment of the block copolymer brush was hydrated and extended, leading to a reduction in the affinity between the peptide and exosome. These results indicate that H5P5-N1500-LTV is suitable for temperature-modulated exosome capture.

**Fig. 6:**
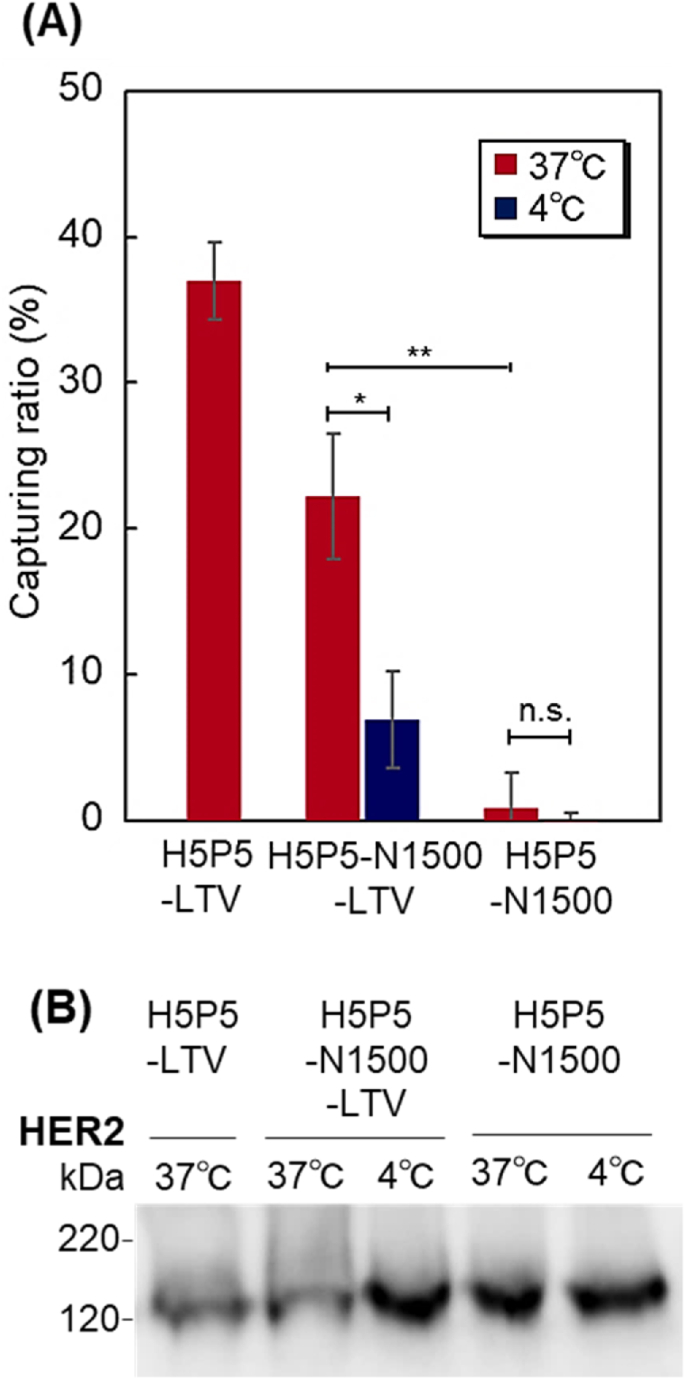
Capturing exosomes on H5P5-LTV, H5P5-N1500-LTV, and H5P5-N1500 beads. (A) Capturing ratio of exosomes derived from SK-BR-3 ​cells. Data are presented as the mean ​± ​standard deviation (n ​= ​3) (∗: *P* ​< ​0.05; ∗∗: *P* ​< ​0.01; n. s.: not significant, n ​= ​3); (B) western blotting of HER2 expression in exosome suspension after capturing exosomes on the beads.

To investigate whether the prepared beads can capture HER2-positive exosomes, western blotting of the suspension was performed after contact between the beads and the exosome suspension (Fig. 6). The suspensions of H5P5-LTV at 37 ​°C and H5P5-N1500-LTV at 37 ​°C exhibited lower amounts of HER2 than those of H5P5-N1500-LTV at 4 ​°C, H5P5-N1500 at 37 ​°C, and H5P5-N1500 at 4 ​°C. These results indicated that H5P5-LTV at 37 ​°C and H5P5-N1500-LTV at 37 ​°C captured HER2-expressing exosome in the suspension, leading to a low amount of HER2 in suspension. H5P5-LTV exposed their peptides to the exosome suspension, leading to the effective capture of exosomes in the suspension. The upper PNIPAAm segment of H5P5-N1500-LTV shrunk at 37 ​°C and exposed the peptide on the bottom P(HEMA-*co*-PgA) segment to the exosome suspension. Thus, exosomes in the suspension were captured on the beads at 37 ​°C, leading to a low amount of HER2 in the suspension. In contrast, the PNIPAAm segment of H5P5-N1500-LTV extended at 4 ​°C and concealed the peptide on the bottom P(HEMA-*co*-PgA) segment in the exosome suspension. Thus, exosomes in the suspension were not effectively captured on the beads at 4 ​°C, leading to a relatively high amount of HER2 in the suspension. H5P5-N1500 did not contain any peptides. Thus, exosomes in the suspension were not effectively captured on the beads at either 37 ​°C or 4 ​°C, leading to a high amount of HER2 in suspension. These results indicate that H5P5-N1500-LTV can capture HER2-expressing exosomes in response to external temperatures by modulating peptide affinity using exosomes.

**Fig. 7:**
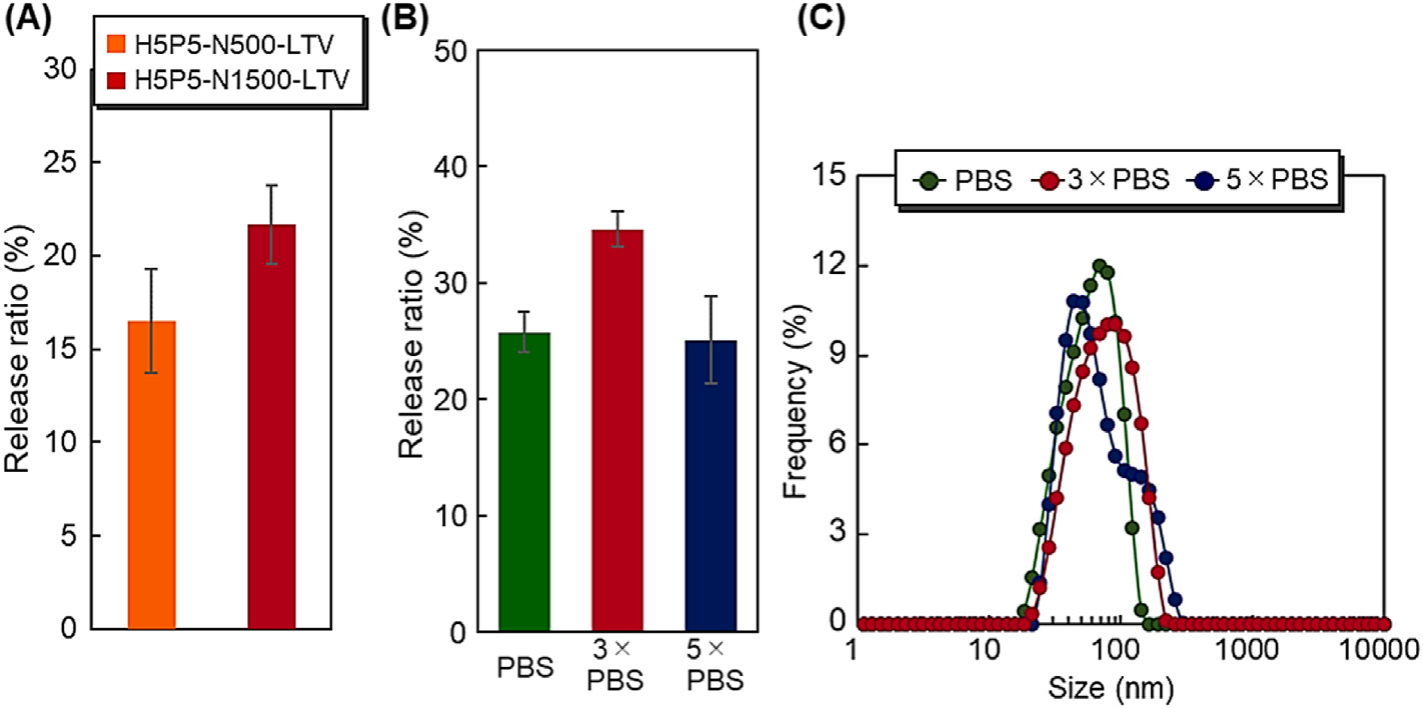
Exosome release on changing the temperature from 37 ​°C to 4 ​°C. (A) Release ratio of exosomes for H5P5-N500-LTV and H5P5-N1500 beads. (B) Release ratio of exosomes for with various concentrations of eluent and (C) Diameter distribution of the released exosomes. Data are presented as the mean ​± ​standard deviation (n ​= ​3).

The release of captured exosomes from the beads was investigated by changing the temperature from 37 ​°C to 4 ​°C using H5P5-N500-LTV and H5P5-N1500-LTV (Fig. 7A). The captured exosomes were released from the copolymer-grafted beads by reducing the temperature from 37 ​°C to 4 ​°C. This was because the PNIPAAm segment of the copolymer was extended by lowering the temperature attributed to the hydration of PNIPAAm, leading to a reduced affinity between the exosome and peptides in the bottom segment of the copolymer brush. H5P5-N1500-LTV exhibited a higher release ratio than H5P5-N500-LTV. This difference was attributed to the length of the PNIPAAm segment in the bottom copolymer brush. H5P5-N1500-LTV has a relatively long PNIPAAm chain compared to H5P5-N500-LTV. By reducing the temperature from 37 ​°C to 4 ​°C, the shrinking PNIPAAm segment extended the hydration of PNIPAAm, leading to the release of the captured exosomes from the copolymer-grafted beads. In the case of long PNIPAAm segments, such as H5P5-N1500-LTV, the structural change in the PNIPAAm segment was larger than that in the short PNIPAAm segment. Thus, a larger amount of captured exosomes was released from the copolymer brush in the long PNIPAAm segments.

To investigate the effect of ion concentration of the buffer on exosome release, various concentrations of buffers were used to release exosomes (Fig. 7B). The use of a 3-fold concentration of PBS (3 ​× ​PBS) resulted in a larger release ratio of exosomes than that obtained with PBS. This is probably because the ion concentration of 3 ​× ​PBS was appropriate for releasing exosomes, which was attributed to the reduced electrostatic interactions between the peptide and exosomes. In contrast, the use of a 5-fold concentration of PBS (5 ​× ​PBS) resulted in a smaller release ratio of exosomes than that obtained with 3 ​× ​PBS. These results indicated that an excessive concentration of PBS is unsuitable for exosome release. This is probably due to the excessive concentration of PBS leading to the shrinkage of the PNIPAAm segment, which is attributed to the salting-out effect. Thus, the number of exosomes released upon the extension of PNIPAAm was reduced by a high concentration of PBS. The diameter of the exosomes obtained from the copolymer brush was observed to confirm the maintenance of exosome structure through a temperature-modulated release process (Fig. 7C). Size distribution of the released exosomes was 30–100 ​nm, indicating that the released exosomes maintained their structure after being released from the copolymer brush. In addition, diameter distribution was not significantly changed after changing the concentration of PBS, indicating that the released exosomes did not collapse in the range of PBS concentrations.

These results indicate that the temperature-modulated capture and release of the targeted exosome can be performed using the developed peptide-conjugated copolymer brush-grafted silica beads by simply changing the temperature. In comparison with other exosome separation methods (Table S1), the developed methods are cost effective, have low contamination risk with reagents, do not require special equipment, and have large and scalable sample capacity. Thus, the prepared beads could be useful in a convenient exosome isolation method in diagnostic applications or in methods to prepares exosome as drug delivery carriers.

To further improve the developed temperature-modulated exosome capturing system, a bead-packed column would be an effective approach. The prepared copolymer and peptide modified beads are packed into the column, the exosome suspension is introduced into the column at 37 ​°C where the exosome effectively makes contact with the peptide-modified copolymer brush, and then, cooled buffer solution is flowed into the column. The exosome effectively detaches from the copolymer brush because the shear flow of the buffer solution enhances this detachment, leading to an increased recovery rate. Furthermore, the developed copolymer brush with peptide can be applied to silica-coated magnetic beads by substituting base materials from silica beads to the silica-coated magnetic beads. The copolymer-grafted silica-coated magnetic beads can be easily manipulated by magnetic force, leading to increased simplicity of the procedure.

## 4 Conclusions

Temperature-modulated exosome-capturing materials were developed using P(HEMA-*co*-PgA)-*b*-PNIPAAm brush-grafted silica beads with affinity peptides through tandem ATRP for copolymer modification on silica beads and click reaction for peptide modification to the copolymer brush. The copolymer brush P(HEMA-*co*-PgA)-*b*-PNIPAAm brushes with affinity peptides were successfully modified on silica bead surfaces through the two steps of ATRPs and the subsequent click reaction. Exosomes possessing HER2 protein could be captured on the prepared copolymer-grafted beads at 37 ​°C because of the shrinkage of the PNIPAAm segment, leading to enhanced affinity between exosomes and peptides at the bottom segment of the copolymer. Upon reducing the temperature from 37 ​°C to 4 ​°C, the captured exosomes were released from the beads because the PNIPAAm segment extended, leading to a reduced affinity between the peptide and the exosome. These results indicate that the prepared copolymer brush-grafted silica beads with peptides could selectively capture exosomes that are released by temperature modulation. Thus, the developed copolymer-grafted beads with peptides would be useful for the separation of exosomes using simple procedures such as temperature modulation for diagnosis or utilization as drug carriers in drug delivery.

## Credit author statement

**Kenichi Nagase**: Conceptualization, Writing - Review & Editing, Supervision, **Kaichi Yamazaki**: Methodology, Investigation, **Yutaro Maekawa**: Conceptualization, Methodology, Investigation, **Hideko Kanazawa**: Conceptualization, Supervision.

## Funding

This work was partially supported by Grant-in-Aid for Scientific Research [grant numbers 19H02447, 20H05233, and 22H04560] from the 10.13039/501100001691Japan Society for the Promotion of Science, Japan.

## Declaration of competing interest

The authors declare that they have no known competing financial interests or personal relationships that could have appeared to influence the work reported in this paper.

## Data Availability

Data will be made available on request.
