# Supplementary material for: Thermoresponsive bio-affinity interfaces for temperature-modulated selective capture and release of targeted exosomes
Source: Mater Today Bio. 2022 Dec 11;18:100521. doi: 10.1016/j.mtbio.2022.100521 (PMC9800632; doi:10.1016/j.mtbio.2022.100521)
Supplement: Multimedia component 1 [file mmc1.docx]

*Supplementary Materials*

Thermoresponsive bio-affinity interfaces for the temperature-modulated selective capture and release of targeted exosomes

Kenichi Nagase*^,a^ Kaichi Yamazaki ^a^, Yutaro Maekawa ^a^, and Hideko Kanazawa ^a^

^a^ Faculty of Pharmacy, Keio University, 1-5-30 Shibakoen, Minato, Tokyo 105-8512, Japan

*Corresponding author Tel: +81-3-5400-1378; Fax: +81-3-5400-1378

E-mail: nagase-kn@pha.keio.ac.jp or nagase.kenichi@keio.jp

**Table S1** Comparison of exosome isolation techniques.

| Technique | Principle | Products | Advantage | Disadvantage | Ref. |
| --- | --- | --- | --- | --- | --- |
| Ultra-centrifugation | Density-, size-, and shape-based separations | Ultra-centrifugation apparatus | Reduced cost and contamination risks with separation reagents; large sample capacity | High equipment cost; cumbersome; labor intensive; low portability | [1-4] |
| Size-based separation | Size-based separation | Size exclusion chromatography; filtration device | Does not require special equipment; good portability of equipment | Shear stress-induced  deterioration | [5-7] |
| Exosome Precipitation | Change in the solubility or dispersibility  of exosomes using  water-excluding polymers | Exosome isolation kit (water exclusion polymer) | Does not require special equipment, large and scalable sample capacity | Co-precipitation of other non-exosomal contaminants; requires pre-and post-cleanup. | [2, 6, 8] |
| Immunoaffinity capture-based separation | Specific  interaction between the antigens of exosomes and  immobilized antibodies | Affinity magnetic beads | High selectivity | High reagent cost; low efficiency | [2, 3, 9] |
| Microfluidics | Immunoaffinity, size,  and density | Microfluidic device | High efficiency and  cost-effective | Low sample capacity | [1, 6, 10] |

**S.1 Materials**

*N*-Isopropylacrylamide (NIPAAm) was obtained from KJ Chemicals Corporation (Tokyo, Japan). Tris(2-aminoethyl)amine (TREN), 2-propanol, hydrochloric acid, acetone, dimethyl sulfoxide (DMSO), toluene, ascorbic acid, copper (II) chloride (CuCl_2_), *n*-hexane, copper (II) sulfate pentahydrate (CuSO_4_), and sodium ascorbate were purchased from Fujifilm (Osaka, Japan). Tris[(2-dimethylamino)ethyl]amine (Me_6_TREN) was obtained using TREN [11]. Tris[(1-benzyl-1H-1,2,3-triazol-4-yl)methyl]amine (TBTA), *α*-chloro-*p-*xylene, and 2-hydroxyethyl methacrylate (HEMA) were obtained from Tokyo Chemical Industry (Tokyo, Japan). Propargyl acrylate (PgA) and ((chloromethyl)phenylethyl)-trimethoxysilane (CPTMS) were purchased from Sigma-Aldrich (St. Louis, MO, USA) and Gelest (Morrisville, PA, USA), respectively. Pierce Micro BCA™ Protein Assay kit, Total Exosome Isolation kit (TEI), penicillin-streptomycin solution (PS), and McCoy’s 5A (Modified) Medium were purchased from Thermo Fisher Scientific (Waltham, MA, USA). HeLa cells and SK-BR-3 cells were purchased from the RIKEN Cell Bank (Tsukuba, Japan) and American Type Culture Collection (Manassas, VA, USA), respectively. RIPA buffer (10×), polyoxyethylene sorbitan monolaurate (Tween-20), skim milk, and WB Stripping Solution were obtained from Nacalai Tesque (Kyoto, Japan). Goat anti-mouse IgG (HRP), goat anti-rabbit IgG (HRP), and anti-CD63 mouse monoclonal antibody were obtained from Abcam (Cambridge, MA, USA). ECL™ Prime Western Blotting Detection reagent and anti-HER2 rabbit monoclonal antibody were obtained from GE Healthcare (Boston, MA, USA) and Cell Signaling Technology (Danvers, MA, USA), respectively. Affinity peptides N_3_-Gly-Gly-Gly-Leu-Thr-Val-Ser-Pro-Trp-Tyr and N_3_-Gly-Gly-Gly-Lys-Cys-Cys-Tyr-Ser-Leu were obtained from GL Biochem (Shanghai, China). Silica beads (SUNSPHERE® NP-30; diameter: 4.0 μm; surface area: 40 m^2^/g; non-porous) were kindly provided by AGC Si-Tech (Fukuoka, Japan).

**S.2 Calculation of grafted initiator, polymer, and peptide**

To estimate the amounts of initiator, polymer, and peptide, CHN elemental analysis of the prepared beads was conducted using an elemental analyzer (PE2400-II; PerkinElmer, Waltham, MA, USA). The carbon composition of the prepared silica beads was used to estimate the amounts of initiator, polymer, and peptide. The amount of initiator used was calculated as follows:

$\frac{\%C_{I}}{\%C_{I}\left( calcd \right) \times\left( 1 - \%C_{I}/\%C_{I}(calcd) \right) \times S}$ (1)

where *%C_I_* is the increase in carbon percentage after the silanization reaction of CPTMS, *%C_I_(calcd)* is the theoretical carbon percentage of the CPTMS, and *S* is the surface area of the silica beads (40 m^2^/g).

The amount of P(HEMA-*co*-PgA) grafted onto the silica beads was calculated as follows:

$\frac{\%C_{HP}}{\%C_{HP}\left( calcd \right) \times\left( 1 - \%C_{HP}/\%C_{HP}\left( calcd \right)-\%C_{I}/\%C_{I}(calcd) \right) \times S}$ (2)

where *%C_HP_* is the increase in carbon percentage through the first ATRP, and *%C_HP_(calcd)* is the theoretical carbon percentage of the P(HEMA-*co*-PgA).

The amount of PNIPAAm grafted onto the silica beads was calculated as follows:

$\frac{\%C_{N}}{\%C_{N}\left( calcd \right) \times\left( 1 - \%C_{N}/\%C_{N}\left( calcd \right)- \%C_{HP}/\%C_{HP}\left( calcd \right)-\%C_{I}/\%C_{I}(calcd) \right) \times S}$ (3):

where *%C_N_* is the increase in carbon percentage through the second ATRP, and *%C_N_(calcd)* is the theoretical carbon percentage of the PNIPAAm.

The amount of the grafted peptide was calculated as follows:

$\frac{\%C_{P}}{\%C_{P}\left( calcd \right) \times\left( 1 - \%C_{P}/\%C_{P}\left( calcd \right)- \%C_{N}/\%C_{N}\left( calcd \right)- \%C_{HP}/\%C_{HP}\left( calcd \right)-\%C_{I}/\%C_{I}(calcd) \right) \times S}$ (4)

where *%C_P_* is the increase in carbon percentage after the click reaction, and *%C_P_(calcd)* is the theoretical carbon percentage of peptide.


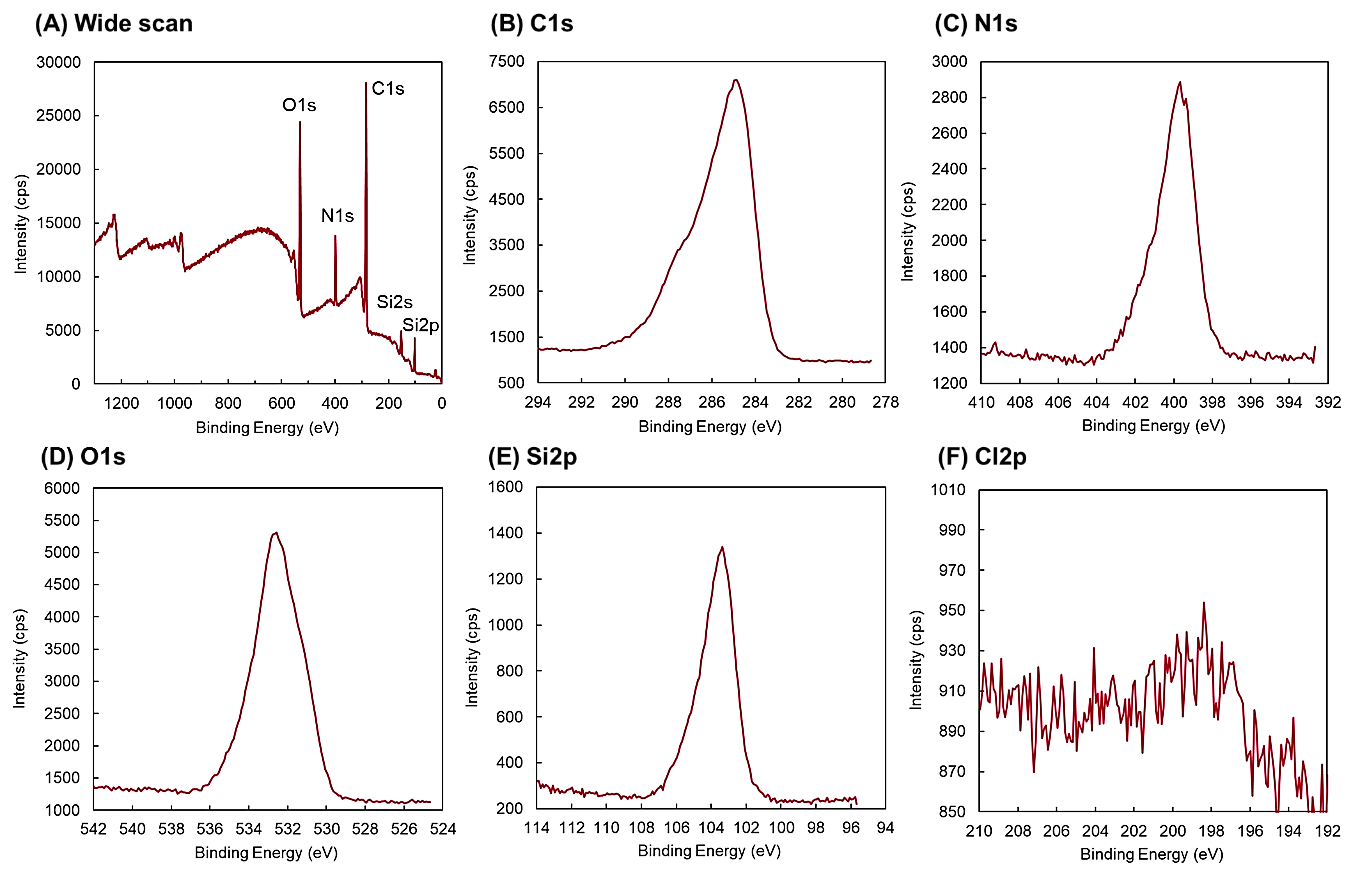


**Fig. S1** X-ray photoelectron spectroscopic spectra of P(HEMA-co-PgA)-b-PNIPAAm with GGGLTVSPWY (H5P5-N500-LTV)-modified silica beads, at a take-off angle of 90°.


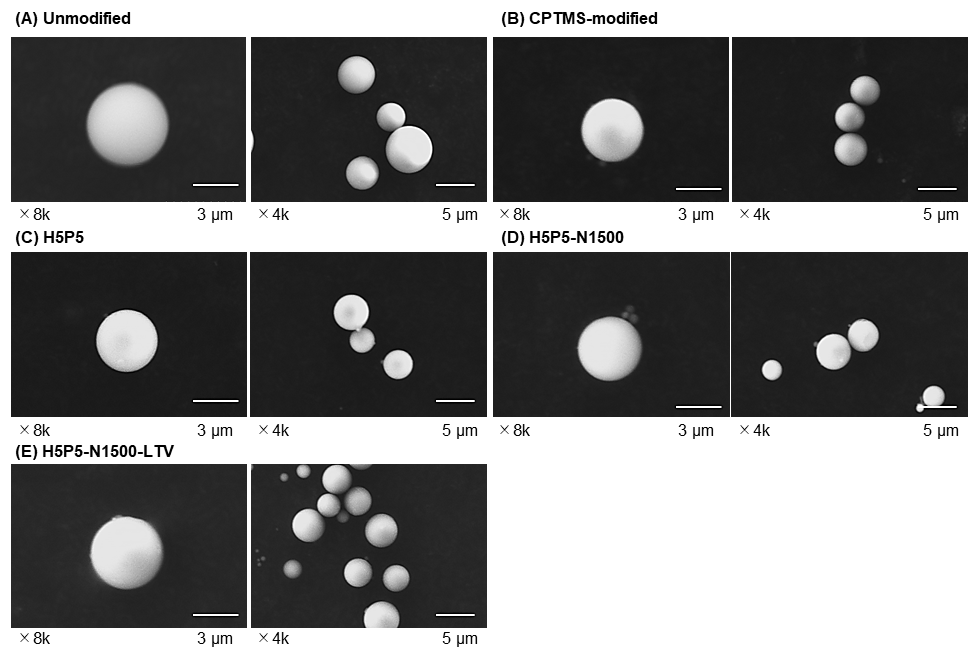


**Fig. S2** Scanning electron microscopy images of the prepared beads. (A) Unmodified, (B) CPTMS-modified, (C) H5P5, (D) H5P5-N1500, and (E) H5P5-N1500-LTV beads.


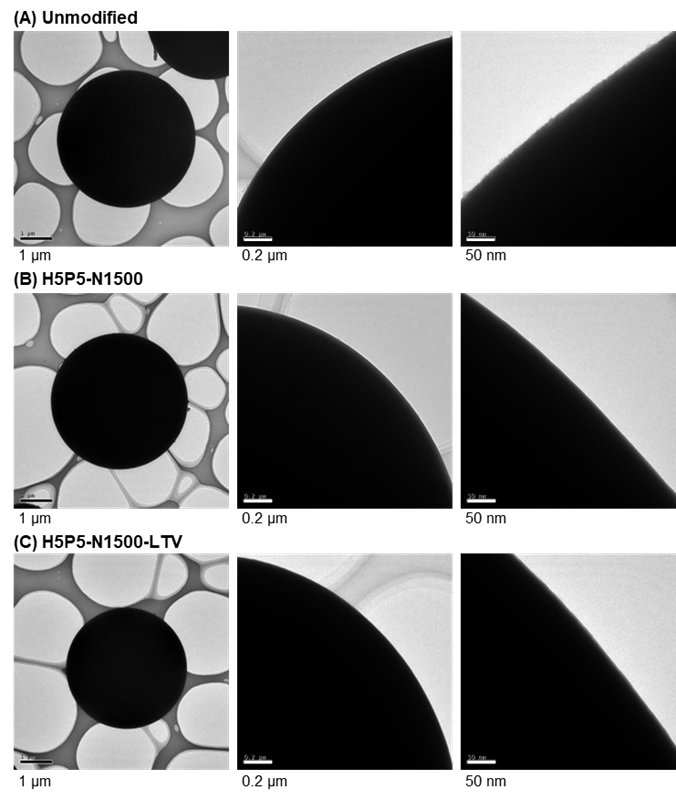


**Fig. S3** Transmission electron microscopy images of the prepared beads. (A) Unmodified, (B) H5P5-N1500, and (C) H5P5-N1500-LTV beads.

**Table S2** Molecular weights and polydispersities of the prepared polymers.

| Code | Copolymer type | Monomer conc.  (mM) | *M_n_* ^a)^ | *M_w_* ^a)^ | *M_w_/M_n_* ^a)^ |
| --- | --- | --- | --- | --- | --- |
|  |  |  |  |  |  |
| H9P1 | P(HEMA-*co*-PgA) | 200 | 12300 | 15400 | 1.25 |
| H5P5 | P(HEMA-*co*-PgA) | 200 | 9000 | 13400 | 1.48 |
| N500 | PNIPAAm | 500 | 11600 | 15000 | 1.29 |
| N1500 | PNIPAAm | 1500 | 22000 | 35400 | 1.61 |

a) Determined *via* gel permeation chromatography using *N,N*-dimethylformamide with 50 mM lithium chloride as the mobile phase.


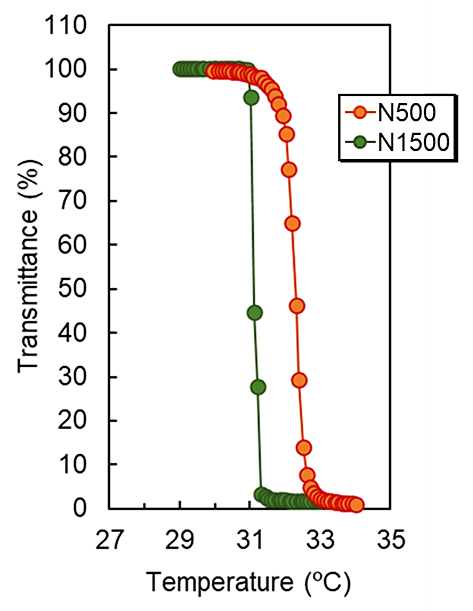


**Fig. S4** Phase transition behavior of PNIPAAm segments of different lengths in phosphate-buffered saline.

**
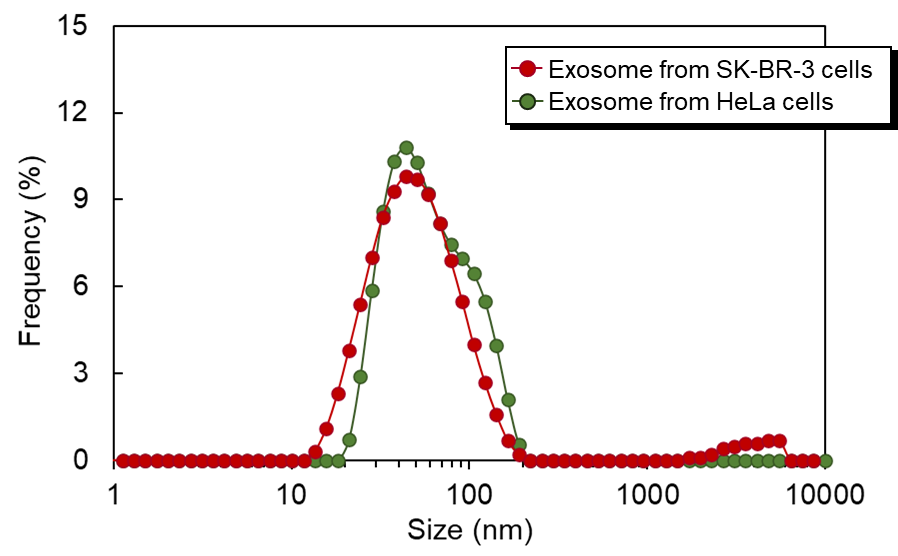
**

**Fig. S5** Particle size of exosomes collected from SK-BR-3 cells and HeLa cell culture media.

**
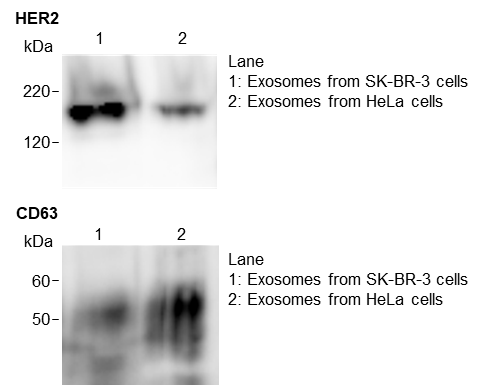
**

**Fig. S6** Western blotting of exosomes collected from SK-BR-3 cells and HeLa cell culture media.

**References**

[1] P. Li, M. Kaslan, S.H. Lee, J. Yao, Z. Gao, Progress in Exosome Isolation Techniques, Theranostics 7(3) (2017) 789-804.

[2] I.L. Colao, R. Corteling, D. Bracewell, I. Wall, Manufacturing Exosomes: A Promising Therapeutic Platform, Trends Mol. Med. 24(3) (2018) 242-256.

[3] P. Akuma, O.D. Okagu, C.C. Udenigwe, Naturally Occurring Exosome Vesicles as Potential Delivery Vehicle for Bioactive Compounds, Frontiers in Sustainable Food Systems 3 (2019).

[4] B.J. Benedikter, F.G. Bouwman, T. Vajen, A.C.A. Heinzmann, G. Grauls, E.C. Mariman, E.F.M. Wouters, P.H. Savelkoul, C. Lopez-Iglesias, R.R. Koenen, G.G.U. Rohde, F.R.M. Stassen, Ultrafiltration combined with size exclusion chromatography efficiently isolates extracellular vesicles from cell culture media for compositional and functional studies, Scientific Reports 7(1) (2017) 15297.

[5] S. Inamdar, R. Nitiyanandan, K. Rege, Emerging applications of exosomes in cancer therapeutics and diagnostics, Bioengineering & Translational Medicine 2(1) (2017) 70-80.

[6] K. Abhange, A. Makler, Y. Wen, N. Ramnauth, W. Mao, W. Asghar, Y. Wan, Small extracellular vesicles in cancer, Bioactive Materials 6(11) (2021) 3705-3743.

[7] K. Sidhom, P.O. Obi, A. Saleem, A Review of Exosomal Isolation Methods: Is Size Exclusion Chromatography the Best Option?, Int J Mol Sci, 2020.

[8] S. Gurunathan, M.-H. Kang, M. Jeyaraj, M. Qasim, J.-H. Kim, Review of the Isolation, Characterization, Biological Function, and Multifarious Therapeutic Approaches of Exosomes, Cells, 2019.

[9] S.M. Patil, S.S. Sawant, N.K. Kunda, Exosomes as drug delivery systems: A brief overview and progress update, Eur. J. Pharm. Biopharm. 154 (2020) 259-269.

[10] S.Z. Shirejini, F. Inci, The Yin and Yang of exosome isolation methods: conventional practice, microfluidics, and commercial kits, Biotechnol. Adv. 54 (2022) 107814.

[11] M. Ciampolini, N. Nardi, Five-Coordinated High-Spin Complexes of Bivalent Cobalt, Nickel, andCopper with Tris(2-dimethylaminoethyl)amine, Inorg. Chem. 5(1) (1966) 41-44.
